# Ultrasound elastography in children — nice to have for scientific studies or arrived in clinical routine?

**DOI:** 10.1186/s40348-022-00143-1

**Published:** 2022-06-06

**Authors:** Hans-Joachim Mentzel, Katja Glutig, Stephanie Gräger, Paul-Christian Krüger, Matthias Waginger

**Affiliations:** Section of Pediatric Radiology, Department of Radiology, University Hospital Jena, Am Klinikum 1, 07747 Jena, Germany

**Keywords:** Ultrasound elastography, Acoustic radiation force impulse, Shear wave elastography, Transient elastography

## Abstract

Ultrasound elastography (USE) is a modality that in addition to fundamental B-mode, Doppler, and contrast-enhanced sonography is suitable to make qualitative and quantitative statements about the stiffness of tissues. Introduced more than 20 years ago in adults, USE becomes now a diagnostic tool also in children. The aim of this paper is to describe current available techniques for USE in children. The significance for routine use in children is shown, and further interesting applications are reported.

## Introduction and history

Ultrasound is the central imaging method in pediatric diagnostics caused by the neglectable risk profile and high availability. In addition to fundamental B-mode sonography, Doppler modalities and contrast-enhanced ultrasound elastography (USE) were introduced more than 20 years ago into the clinical routine in adults increasing the diagnostic power of ultrasound examinations.

In medicine, palpation is a standard diagnostic tool in clinical examination and has always been used to differentiate benign and malignant lesions using the criteria of size, displaceability, compressibility, hardness, and consistency. Unfortunately, only superficial structures are accessible to palpation. Furthermore, palpation is a subjective method and strongly dependent on the experience of the physician. These limitations can be overcome with elastography, a procedure developed for both magnetic resonance (MR) and ultrasound. While MR elastography has so far not been able to establish in clinical routine due to considerable expenditure, USE plays an increasing role.

Elastography is used to measure noninvasively the internal tissue deformation that results after application of a force. According to the English ophthalmologist and physicist Thomas Young (1773–1829), elasticity is described using the modulus elasticity E, which is calculated from the ratio of tension to stretching of the tissue. The extent of relative changes in length when an external or internal force is applied depends on the elastic properties. Young’s modulus for diamond as the hardest material is 1220 GPa (Gigapascal), healthy soft tissue is between 0.5 and 70 kPa (Kilopascal), and healthy liver tissue is between 0.4 and 6 kPa [54].

Focal and diffuse findings of tissues could be described in more detail in terms of their elasticity. Starting with the evaluation of liver diseases in adults at the end of the 1990s, the horizon of elastography has subsequently expanded significantly. It was used to asses classic malignancies (e.g., breast cancer, prostate cancer) but also to evaluate superficial organs such as the thyroid gland and their focal lesions. Guidelines about the use of elastography in adults were published and updated by the European Federation of Societies for Ultrasound in Medicine and Biology (EFSUMB) and the World Federation for Ultrasound in Medicine and Biology (WFUMB) in adults for hepatic and non-hepatic applications [13, 22, 48]. Up to now, there are only few reports about features of elastography and techniques in children ([14], Mentzel 2020 [42]).

## Technical background

**Fig. 1:**
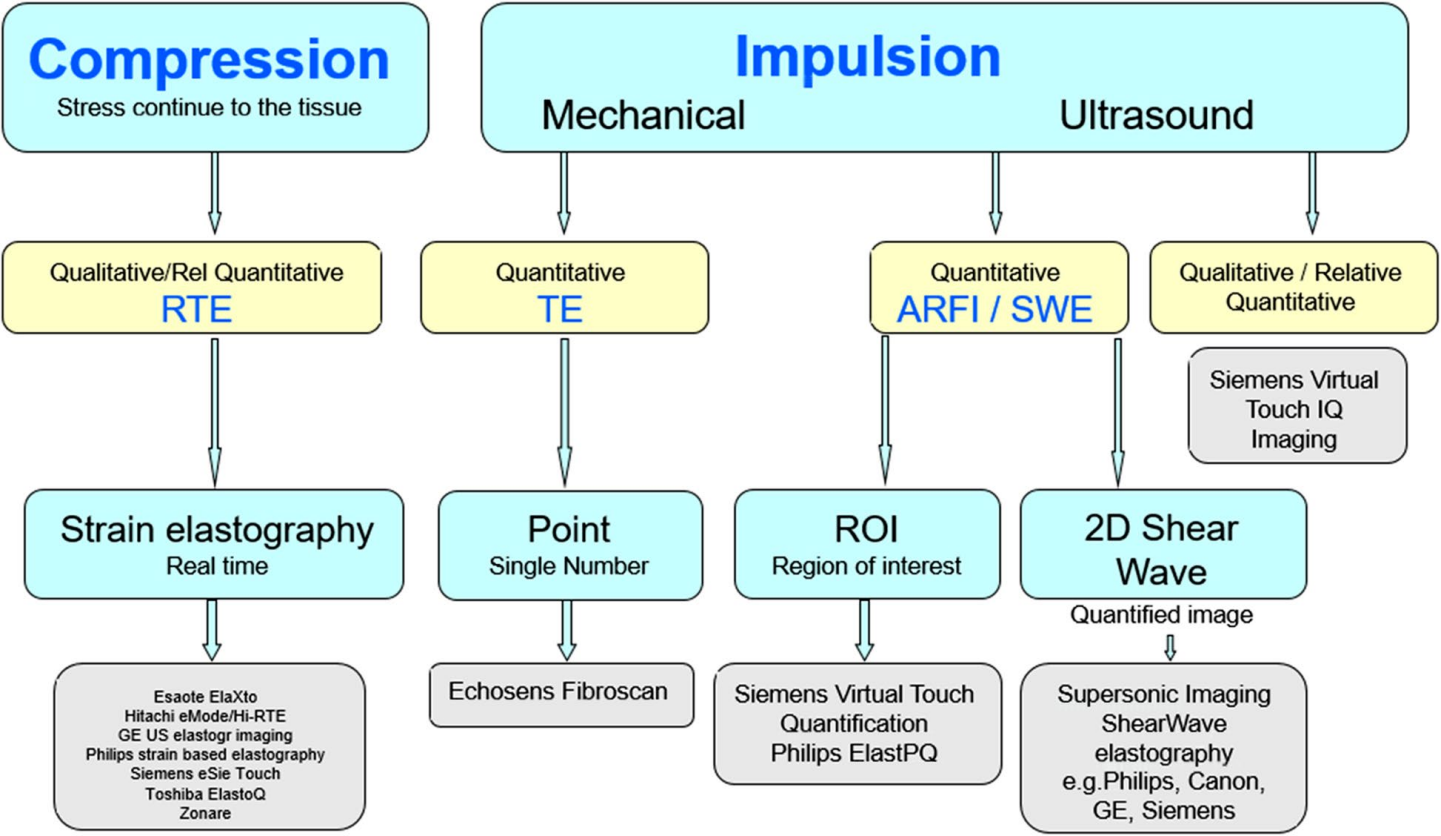
Various methods of ultrasound elastography. All use the measurement of deformation in response to an applied stress or force given by compression or creation of a push pulse (RTE, real-time elastography; TE, transient elastography; ARFI, acoustic radiation force impulse; SWE, shear wave elastography; IQ, image quantification; ROI, region of interest; 2D, two dimensional; GE US, general electric ultrasound; Q, quantification)

The method of static elastography is based on the work of Ophir et al. who published elastograms based on the correlation analysis of tissue displacement under pressure [46]. At the beginning of the new century, dynamic elastography was developed superimposing elasticity values in color on the fundamental B-mode image in real time (real-time elastography, RTE). The strain image results from the different shifts of the echo frequency pattern under mechanical pressure (external compression, internal pulsation, breathing movement) on the analyzed tissue, with elastic tissues approaching the frequency peaks, while with stiff tissues, they remain at a constant distance. Rigid structures are usually shown in blue, intermediate deformable tissue parts are shown in yellow-green, and easily deformable tissue parts are shown in orange-red. Strain elastography provides a color-coded, qualitative result. The strain ratio can be used to estimate at least a ratio of the stiffness of the region of interest (ROI) to the surrounding area. Dynamic USE using shear waves is based on a focused impulse that propagates as a shock wave with a propagation speed of 1540 m/s into the tissue and leads to a compression (virtual touch). Compression is followed by the release of shear waves during the relaxation process. Shear waves propagate according to the rigidity of the environment. The speed of the shear waves (around 1–10 m/s) can be evaluated by a second ultrasonic pulse (acoustic radiation force impulse, ARFI) [21, 44]. Transient elastography (TE) with FibroScan© (Echosens, Paris, France) developed for liver elastography uses both ultrasound around 5 MHz and shear waves with 50 Hz generated by a defined external impulse vibrations. The result is given in kPa as an average of ten subsequent measurements [47]. Disadvantages of this well-standardized procedure are the lack of image control where the pulse is placed and the limitation in obese patients as well as in cases of ascites. ARFI is now widespread and available on most modern ultrasound devices with conventional transducers. A distinction has to be made between ARFI imaging as a qualitative method with representation of tissue deformation as a two-dimensional image and ARFI quantification (e.g., virtual touch quantification, VTQ, Siemens; Elast PQ, Philips) as quantitative tool (point shear wave elastography, pSWE). The measurement area is defined and placed as region of interest by means of B-mode imaging. In the past, ARFI has been expanded to include shear wave elastography (2D-SWE), in which the impulses are applied to a quantification box (Q-Box) at different tissue depths (e.g., supersonic shear imaging, SSI, Aix-en-Provence, France; virtual touch imaging quantification, VTIQ, Siemens, Germany; SWE, Philips, Netherlands; 2D-SWE, GE, USA; acoustic structure quantification, ASQ, Canon, Japan). The size of the evaluated region can be varied in real time, and the shear wave velocity (m/s) or the stiffness (kPa) can be specified [51] (Fig. 1).

The various techniques of USE can be compared among each other only with great caution. Measurements with different devices from various manufacturers, even data collected with different transducers on the same device, can hardly be related to one another. This must be taken into account for any follow-up checks at time intervals. Standardization of one’s own approach is required.

## Clinical established applications

**Fig. 2:**
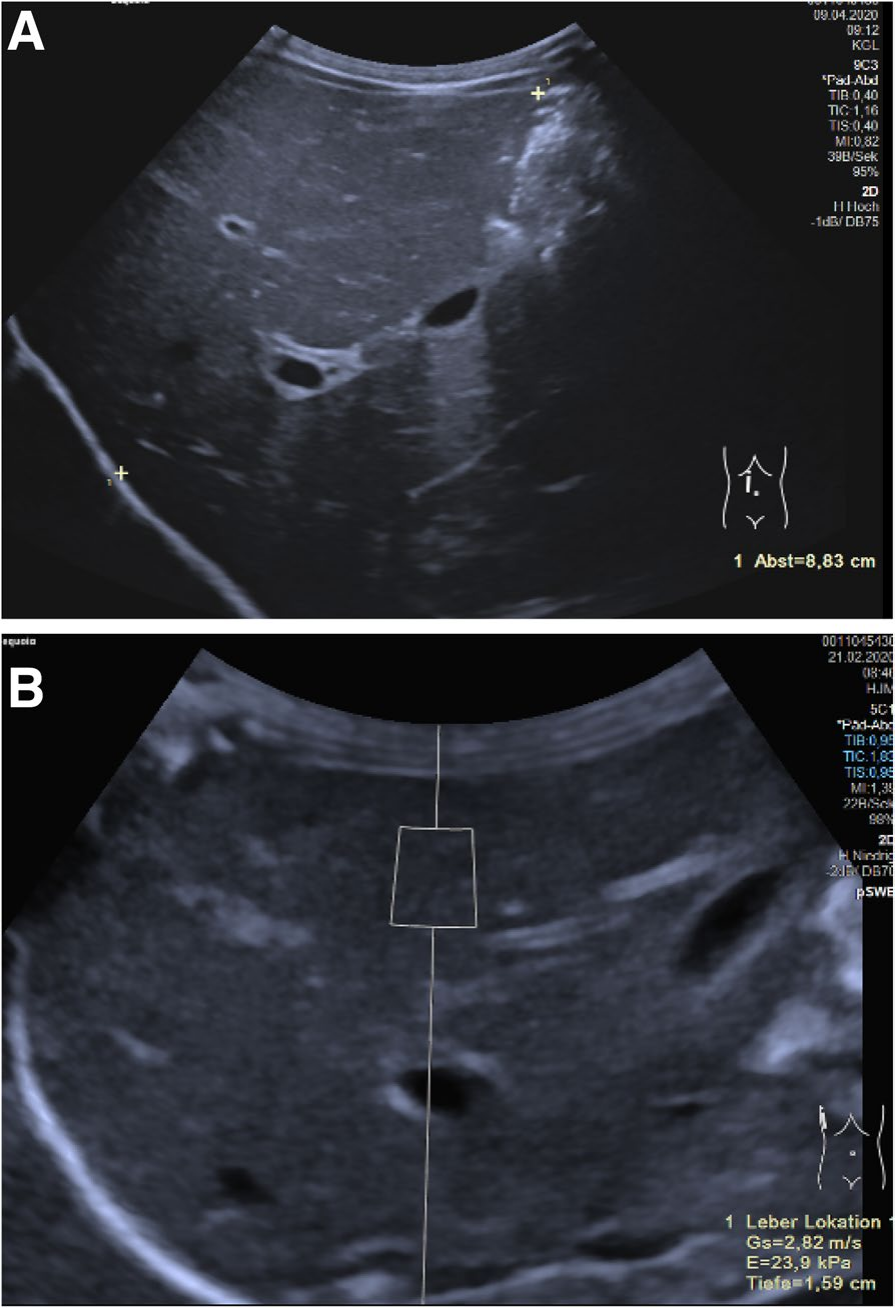
Four-month old male infant with hepatosplenomegaly and cholestasis. Following sonography and elastography biopsy indicated. The result showed Niemann-Pick disease type A associated with liver fibrosis and reduced synthetic capacity of the liver. **A** B-mode imaging of the liver in the midclavicular line corresponding to hepatomegaly (Sequoia, Siemens, 5C3 probe). **B** SWE measurement in the right liver lobe. Shear wave speed of 2.82 m/s is significantly increased and has to be assessed in terms of fibrosis (Sequoia, Siemens, 5C1 probe)

The liver is generally the organ most frequently evaluated by elastography. In addition to some reference value studies in children [41, 43], there are numerous clinical studies published on detecting and grading liver fibrosis as well as on diagnosis and treatment follow-up of biliary atresia in childhood ([16, 32]; Kim JR et al. [39];). Liver stiffness data can be expressed in kPa using TE or in m/s using pSWE or 2D-SWE. The software in modern ultrasound devices allows for conversion of m/s to Young’s modulus kPa for SWE. To assess diffuse liver changes, the right lobe of the liver is usually examined in segment 7. An intercostal access in a supine position with the right arm in extension gives more reproducible data than the evaluation of the left liver lobe [14]. Breath-hold minimizes measurement variability but can be difficult in children below the age of 5 years. Fast 2D-SWE evaluation with free-breathing gives results similar to breath-holding [37]. The liver should not move during the measurement. Region of interest is placed perpendicular to 2 cm subcapsular in the liver. It has proven useful to acquire 10 measurements and to use the median for further assessment [45]. In addition to age and gender, factors influencing liver stiffness are the positioning of the transducer and the region of interest as well as the blood flow to the liver, which depends on the time of last meal. The best reliability in the assessment of the liver stiffness is in examinations using 2D-SWE compared to pSWE and TE [38, 43]. There are promising USE studies for the evaluation of cystic fibrosis-related liver diseases (CFLD) [25]. A cutoff value of 1.27 m/s for CFLD was published achieving a specificity of 97% [9, 10]. For non-alcohol-related steatohepatitis (NASH), the combination of SWE and laboratory parameters has proven to be a biomarker of the severity of this entity [57]. In biliary atresia cases of post-Kasai liver fibrosis, ARFI can be used to assess liver fibrosis and to guide further treatment decisions like the urgency of liver transplantation [15, 29]. Further USE applications include the evaluation of liver pathology in alpha-1-antitrypsin deficiency, autoimmune hepatitis, veno-occlusive disease, hemochromatosis, Wilson’s disease, autosomal recessive polycystic kidney disease, and monitoring after liver transplantation [14, 28] (Fig. 2). USE plays a fixed role in the assessment of relevant liver pathologies and can be classified as a diagnostic component after clinical examination and standard liver laboratory prior to invasive liver biopsy [20, 24]. When evaluating liver elastography values, it should be borne in mind that there is a direct correlation with right-sided cardiac function. Every pathology of the right heart leads to pressure changes in the upstream vascular system, to the hepatic veins via the inferior vena cava. Due to the relatively coarse liver capsule, the changes in vascular pressure also lead to changes in rigidity. Each congestion ultimately leads to increased stiffness in the liver tissue. In children with congenital heart defects, liver stiffness can be used as a prognostic marker for cardiac events [23].

## Other possible applications

While USE of the liver has meanwhile arrived in the clinical routine with children and adolescents, so far, all other applications are only poorly established.

### Spleen

USE of the spleen is usually carried out in connection with examinations of portal hypertension in patients with liver fibrosis or cirrhosis and corresponding underlying diseases such as biliary atresia [63]. The examination is carried out in supine position in an intercostal incision from the lateral side. The region of interest is placed subcapsular at a depth of approximately 2 cm. Reference values given for pSWE (ARFI imaging) and TE can be used for the evaluation of spleen pathologies, e.g., evaluation of early signs of portal hypertension [9, 10, 30, 56].

### Kidney

Caused by complex anatomy and given directionality in the parenchyma with cortex and medulla, the assessment of stiffness with USE is a challenge. Glomerulopathies in particular are a very interesting field for elastography [62]. Changes in the kidney transplant, acute and chronic inflammatory changes, should also have an influence of the parenchymal stiffness. Perfusion, hydration, and diuresis are factors which influence the elasticity of the kidney. Initial studies could show that the rigidity of the renal parenchyma increases with severe dilation [53]. Studies about the influence of vesicoureterorenal reflux on USE values are not without controversy [8]. So far, there are few studies on the use of USE in childhood. In the existing papers, the placement of the measurement region contradicts the standard customary in liver USE that the region of interest (ROI) has to be placed perpendicular to the ultrasound focus. Up to now, there are no standardized guidelines on the use of renal USE [33]. In our experience, the greatest reproducibility of measurement results can be achieved in prone position if, in the longitudinal view of the kidney, the measurement region is placed perpendicular to the focus in the middle of the parenchyma portion near to the transducer. Even then, both the cortex and medulla are visualized within the ROI.

### Thyroid gland

**Fig. 3:**
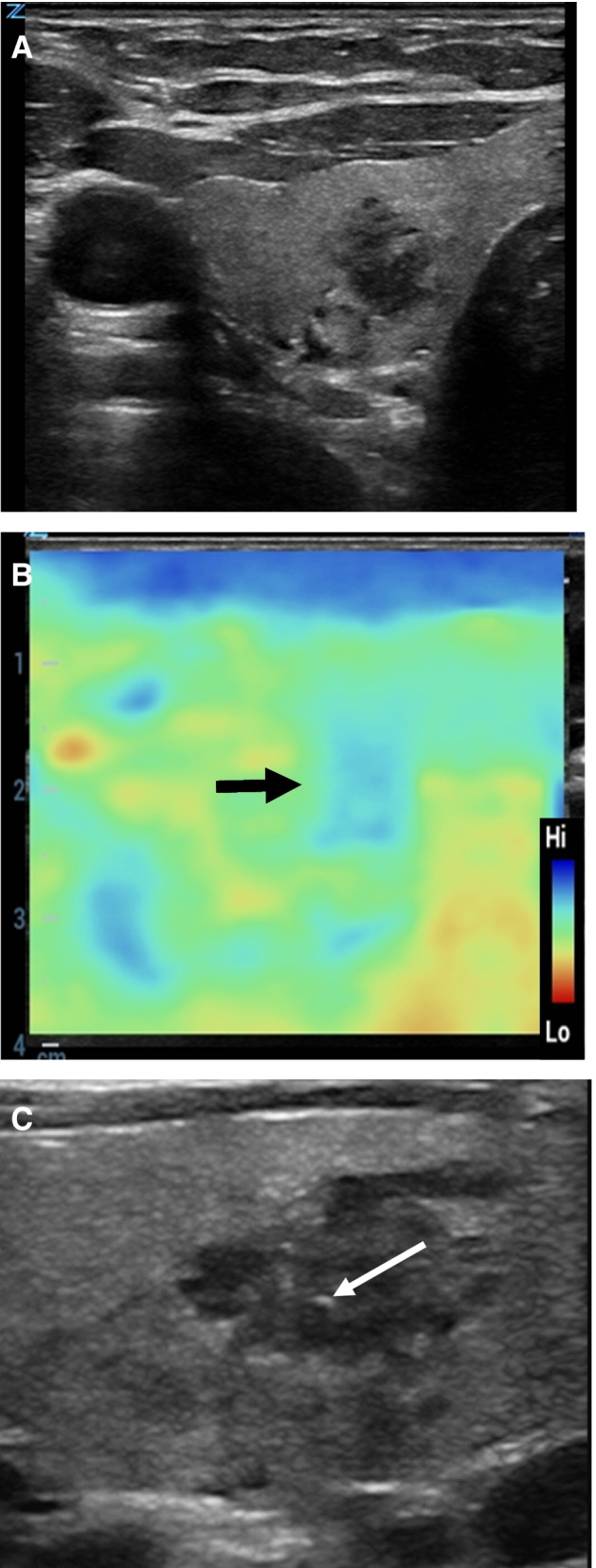
A 15-year-old girl who suffered from nephroblastoma at the age of 12 years. Routine ultrasound control following successful therapy showed a lesion in the right-sided thyroid gland. Histology revealed papillary carcinoma of the thyroid gland, no metastases of the nephroblastoma. **A** B-mode image demonstrates an inhomogeneous hypoechogenic lesion with unsharp borders in the medial part of the right-sided gland (ZS3, Zonare, L14-5 probe). **B** Real-time elastography revealed signs of increased stiffness (blue color, black arrow) of the tumor corresponding to suspected malignancy (ZS3, Zonare, L14-5 probe). **C** B-mode image shows small hyperechogenic bright point with shadowing artefact as a sign of possible calcification (ZS3, Zonare, L20-5 probe)

In the thyroid gland, both the RTE as strain imaging and the SWE in its variations are used. Using RTE, a mechanical impulse administered by the examiner is given by the transducer. Alternatively, pulsation of the carotids can be used as stimulus. Scoring systems have been developed for adults which can be used for the estimation of nodules. Both strain imaging and shear wave elastography were used for autoimmune thyroiditis. In children, a significantly higher shear wave velocity and thus higher stiffness were found compared to children without pathology [4, 11]. Children with type I diabetes mellitus show a significant lower shear wave velocity [49]. USE can be used complementary to ultrasound in predicting thyroid nodules and may be helpful making a decision about fine needle or core biopsy [6, 31] (Fig. 3).

### Lymph nodes

**Fig. 4:**
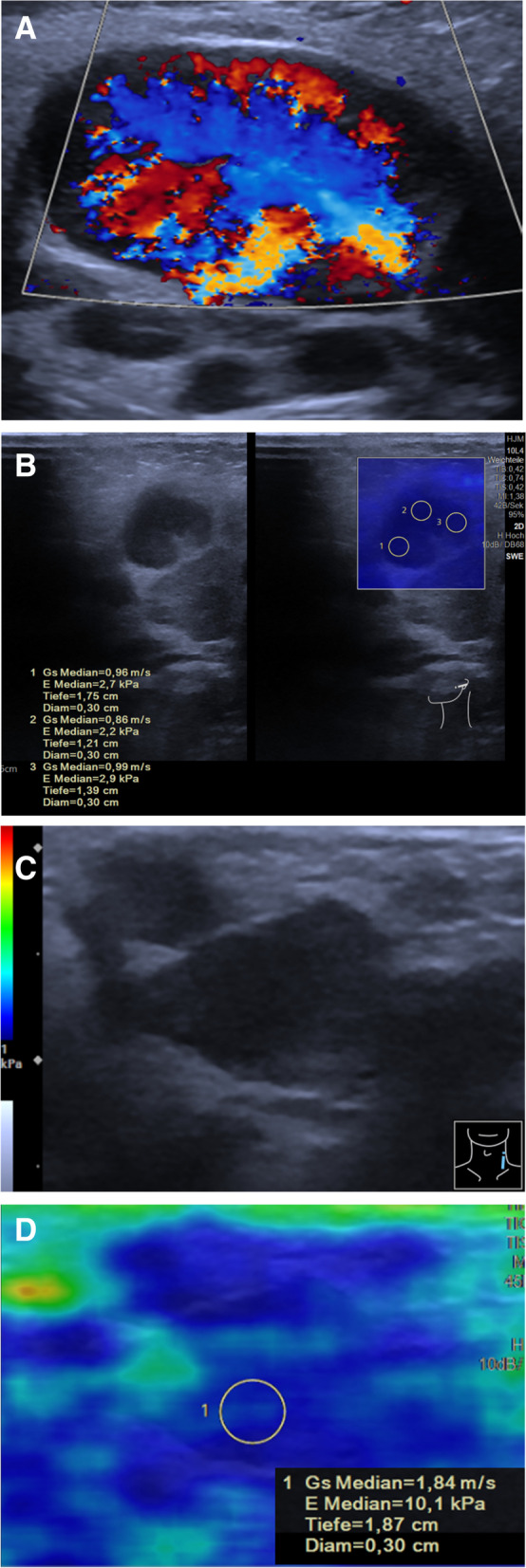
Ultrasound elastography in peripheral cervical lymph nodes. **A** Color Doppler image of a lymph node near the mandible in a 2-year-old male with periapical osteitis caused by caries profunda. There is strong hypervascularization without swelling (Sequoia, Siemens, L14-5 probe). **B** Same case with SWE showing shear wave velocity < 1 m/s (Sequoia, Siemens, L10-4 probe). **C** Color Doppler imaging of a new cervical lymph node in a 14-year-old male with history of Burkitt lymphoma. There was no vascularization detectable (Sequoia, Siemens, L14-5 probe). **D** Same lymph node with SWE showing shear wave velocity of 1.84 m/s corresponding with increased stiffness. Imaging and elastography are suspicious for relapse (Sequoia, Siemens, L10-4 probe)

USE can be added in the ultrasound investigation of superficial lymph nodes using linear transducers. When assessing inflammatory changes in lymph nodes, strain elastography can be used to detect any meltdowns. The problem with USE is the lack of standardization of the examination. Various scoring systems are used to differentiate malignant from benign lymph nodes. Malignant transformed lymph nodes have a predominantly coarse pattern [64]. But, despite good results of USE, it cannot replace biopsy. So, it will be a challenge during the follow-up of children with former malignancy and new lymphadenopathy to differentiate between reactive lymph node enlargement, recurrence, or secondary malignancy [19] (Fig. 4).

### Muscles

The mechanical properties of skeletal muscle include active and passive components related to function, growth, and metabolism. Activity can be characterized by electromyogram. The passive component can be analyzed using USE [61]. Strain imaging has proven itself in muscle elastography, even if there are only few studies on SWE. For strain elastography, a sufficient scoring system of 1–5 was introduced in children ranging from elastic-soft in predominantly red color (score 1), mostly soft in green with 25% red color (score 2), intermediate elastic in predominantly green (score 3) to predominantly hard in green with up to 25% blue (score 4), and finally stiff in blue (score 5) [5]. Using this scaling system, the muscles in children with spastic hemiparesis can be differentiated from healthy children, and, in perspective, the benefit of botulinum toxin therapy with corresponding loosening muscle effect can be monitored [59]. Using shear wave elastography on the gastrocnemic muscle, significantly higher passive stiffness was observed in children with unilateral cerebral palsy [7]. USE can become a valuable method in diagnosis and monitoring of musculoskeletal disorders in children [65].

### Bowel

**Fig. 5:**
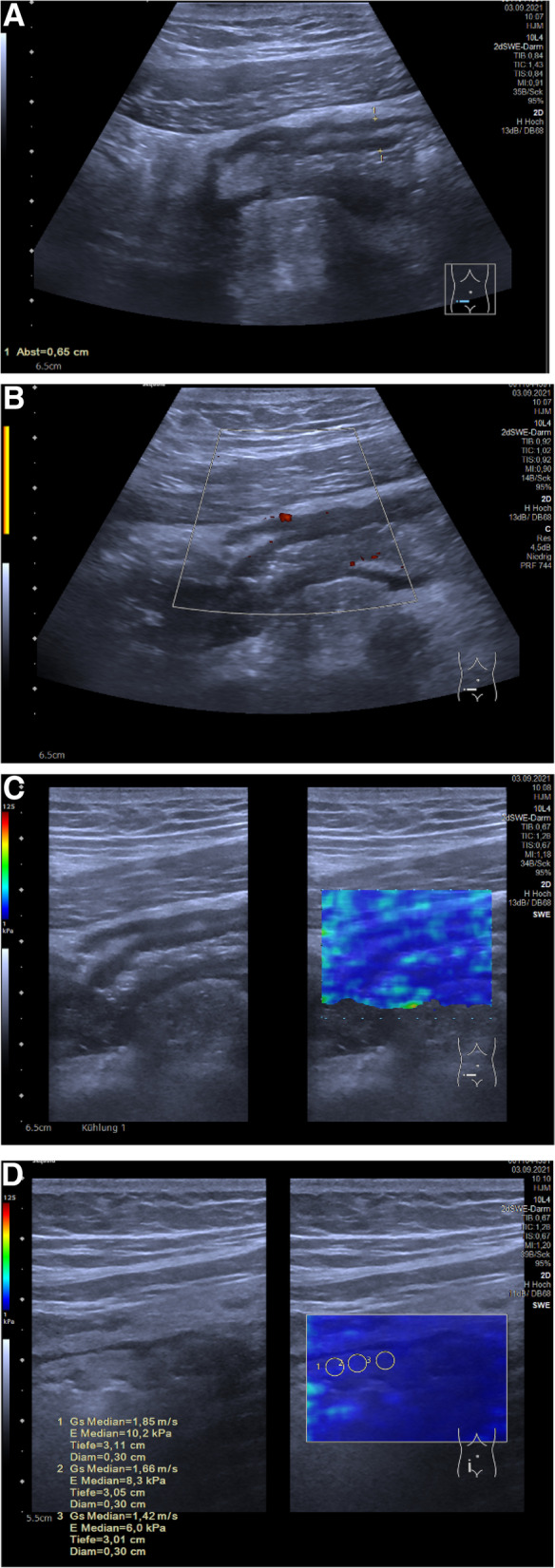
A 16-year-old male with stenosing ileitis in Crohn’s disease. Ultrasound in routine follow-up without acute problems or increasing clinical score. **A** B-mode image revealing increased bowel wall thickness (6.5 mm) (Sequoia, Siemens, L10-4 probe). **B** Power Doppler imaging without any pathological increase in vascularization (Sequoia, Siemens, L10-4 probe). **C** SWE with no pathological qualitative distribution in color scale (Sequoia, Siemens, L10-4 probe). **D** SWE using quantitative evaluation with three regions of interest within the submucosa of the small bowel (median 1.42, 1.66, 1.85 m/s) (Sequoia, Siemens, L10-4 probe) which is lower than published cutoff values for active inflammation [52]

In the gastrointestinal tract, USE may be helpful for differentiating acute inflammation from chronic fibrous alterations of the bowel wall in children with chronic inflammatory bowel diseases (Fig. 5). So far, there are few pilot studies on scoring systems for children in order to estimate inflammatory activity [26]. According to a meta-analysis about 12 papers, USE may be a promising tool supporting evaluation of intestinal strictures in Crohn’s disease as well as the differentiation between fibrosis and inflammation in adults [52]. In addition to the sometimes limited compliance of infants, the frequently lively motility of the intestine which can lead to artifacts is problematic. There are initial studies on strain elastography and SWE in the assessment of acute appendicitis [2, 36].

### Testes

**Fig. 6:**
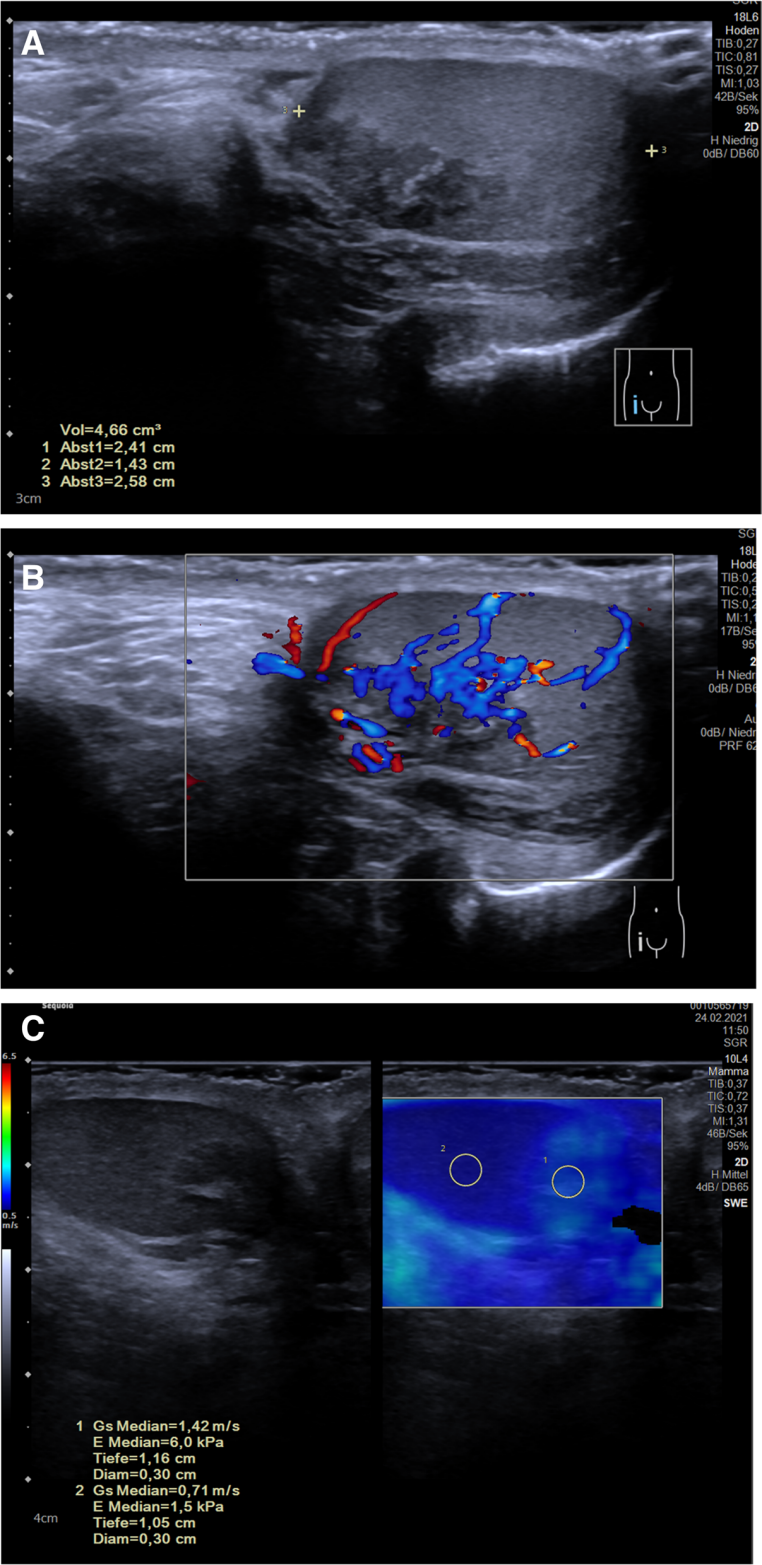
A 13-year-old boy with testicular adrenal rest tumor in adrenogenital syndrome. **A** B-mode image demonstrating hypoechogenic inhomogeneous lesion in the lateral aspect of the testes (Sequoia, Siemens, L18-5 probe). **B** Color Doppler mode showed hypervascularization of the tumor (Sequoia, Siemens, L18-5 probe). **C** SWE demonstrated increased stiffness within the pseudotumor compared to the surrounding physiological tissue (Sequoia, Siemens, L10-4 probe)

The application and possible role of USE to testicular diseases are not well clarified. Up to now, there are few studies regarding the use of SWE for testicular torsion which showed that USE has potential to improve the differentiation between other testicular pathological conditions (e.g., tumor, abscess) and testicular torsion [55] (Fig. 6). In the central part of torsional testis, SWE showed no pathology caused by a mix of edema, hemorrhage, and necrosis. In the border of the twisted testis, SWE values were higher corresponding to increased tissue hardness. But, the twisting sign of the blood vessels (arteria and veins) is the golden standard for the clinical procedure and decision to go in the theater. Furthermore, elastography can be used to evaluate stiffness in undescended testis in comparison with the contralateral descended testes [17, 58] and in patients with microlithiasis who may show increased stiffness [3].

### Brain

Initial reports showed that SWE of the periventricular parenchyma can be successfully performed in premature infants and children with hydrocephalus [12]. The extent to which elastography can be useful to determine increased intracranial pressure has to be verified through studies. Using 2D SWE in preterm infants, less stiffness was measured in the thalamus and in the periventricular parenchyma compared to full-term infants [1]. Anoxic brain injury can be quantified as an increase in stiffness caused by brain edema [12]. One possible application for USE in the future may be as a prognostic marker of neurodevelopmental outcome. Although first reference studies in terms of neonates exist, there are further reference studies necessary [34].

### Lung

Lung ultrasound is imaging of artifacts. Intercostal B-mode imaging of the lung can differentiate A- and B-lines. The number of characteristic reverberation artifacts (B-lines) corresponds to the amount of extravascular lung water. Lung ultrasound surface shear wave elastography has proven itself for the assessment of pulmonary edema in patients with acute congestive heart failure and could show a reduction in shear wave speed suggesting a decrease in stiffness according to positive therapy effects [60]. Actually, USE was used in adults for the diagnosis and management of pneumonia in the COVID-19 pandemic [27]. Up to now, there are no reports about the application of lung USE in children. But, in the case of preterm babies, it could be possible to use elastography for monitoring neonatal respiratory distress syndrome. Further possible applications may be the evaluation of superficial focal lesions as well as monitoring pulmonary edema and interstitial lung disease with pleural/subpleural lesions [35].

## Limitations and safety concern

Limitations lie in the inadequate standardization of the method. There is variability of measurement results using different devices and probes. In the liver, it could be shown that the results of USE are depending on a large number of variables. Compliance of the patient is necessary in most techniques of ultrasound elastography. The approval of ultrasound elastography is limited for use in abdominal, breast, thyroid, small parts, and musculoskeletal examinations. Shear wave elastography is approved by the Food and Drug Administration (FDA) in children and in adults.

The safety of ultrasound is monitored using thermal index (TI) associated with heating effects and mechanical index (MI) associated with cavitation effects. Both indices should be kept as low as possible. Potential risks arising from the energetic ultrasonic push pulse in elastography using acoustic displacement techniques (ARFI, SWE) must be considered [50]. The maximum temperature is at the focus of the evaluated region. The heating increases with the number of pulse sequences and scanning duration. Using a very strong ARFI, a temperature rise of 1.2 °C was estimated in a worst-case scenario (Fahey et al. 2015 [21]). But, in a 2D SWE study on mice, no histologically negative effects were found by SWE [40]. However, some temporary limited effects were observed if the scanning procedure lasted more than 30 min. As mentioned by the EFSUMB, the as low as reasonably achievable (ALARA) principle should be applied when setting the output for ARFI, and scanning time should be kept short [18]. Especially in case of vulnerable tissue (e.g., ovarian or testicular torsion, ischemic brain tissue), caution is necessary.

## Conclusion

Ultrasound elastography which has been known for around 30 years was adopted into clinical routine in adulthood. The existing guidelines on adults may not be immediately transferrable to children [14]. Most of the experience with the use of various ultrasonic elastography methods comes from liver examinations — here, due to existing reference and cutoff value studies, elastography has now in many places become part of the routine in children and adolescents. But, there is still ample research potential for elastography in other organ systems.

## Data Availability

Not applicable.

## Abbreviations

ALARA: As low as reasonably achievable
ARFI: Acoustic radiation force impulse
B-mode: Brightness mode
cm: Centimeter
COVID-19: Coronavirus SARS-CoV-2
E: Elasticity
EFSUMB: European Federation of Societies for Ultrasound in Medicine and Biology
FDA: Food and Drug Administration
GPa: Gigapascal
kPa: Kilopascal
MI: Mechanical index
MR: Magnetic resonance
m/s: Meter per second
pSWE: Point shear wave elastography
ROI: Region of interest
RTE: Real-time elastography
SWE: Shear wave elastography
2D SWE: Two-dimensional shear wave elastography
TE: Transient elastography
TI: Thermal index
USE: Ultrasound elastography
WFUMB: World Federation of Ultrasound in Medicine and Biology
